# Species richness and vulnerability to disturbance propagation in real food webs

**DOI:** 10.1038/s41598-019-55960-8

**Published:** 2019-12-18

**Authors:** Edoardo Calizza, Loreto Rossi, Giulio Careddu, Simona Sporta Caputi, Maria Letizia Costantini

**Affiliations:** 1 Department of Environmental Biology, Sapienza University of Rome, Via dei Sardi 70, 00185 Rome, Italy; 2 National Inter-University Consortium for Marine Sciences (CoNISMa), Piazzale Flaminio 9, 00196 Rome, Italy

**Keywords:** Biodiversity, Ecological networks

## Abstract

A central issue in ecology is understanding how complex and biodiverse food webs persist in the face of disturbance, and which structural properties affect disturbance propagation among species. However, our comprehension of assemblage mechanisms and disturbance propagation in food webs is limited by the multitude of stressors affecting ecosystems, impairing ecosystem management. By analysing directional food web components connecting species along food chains, we show that increasing species richness and constant feeding linkage density promote the establishment of predictable food web structures, in which the proportion of species co-present in one or more food chains is lower than what would be expected by chance. This reduces the intrinsic vulnerability of real food webs to disturbance propagation in comparison to random webs, and suggests that biodiversity conservation efforts should also increase the potential of ecological communities to buffer top-down and bottom-up disturbance in ecosystems. The food web patterns observed here have not been noticed before, and could also be explored in non-natural networks.

## Introduction

The complexity of food webs and the multitude of stressors affecting ecosystems limit our comprehension of how disturbance propagates in ecological communities, hampering biodiversity conservation and management. To cope with such complexity, ecologists have investigated the relationship between the topology of food webs and their stability^1^, seeking to understand whether and how the number of species underlies biodiversity architecture and stability to perturbation^2^. May^3^ argued that in randomly assembled multispecies systems, increasing species richness reduced system stability. This prompted several subsequent studies, which established that the arrangement of food webs with respect to the number of species they contain is not random^4–6^, and that such non-random structures are more stable to perturbation than what is expected by chance^7–10^. Recently, studies have focused on network-level properties of food webs that promote community persistence^9,11^, resilience to perturbations^10^ and resistance to species loss^12–14^. Nevertheless, our understanding of disturbance propagation mechanisms in real ecosystems is still limited. In addition, while food web modelling has fundamentally advanced our understanding of their structure and dynamics, the complexity of the phenomena, the quantity of data involved and the computational skills required have limited the ability to translate such science-based knowledge into practical advice supporting policy formulation^15,16^. Such insight is particularly urgent in a scenario of global change, given the evidence suggesting that both anthropogenic pressure and climate change have the potential to disrupt top-down and bottom-up control mechanisms that regulate ecological communities^17–21^, highlighting the need for management strategies aimed at preserving stable food webs in ecosystems^22,23^.

Within food webs, disturbance can propagate along food chains either from a basal resource towards its consumers and their predators (bottom-up pathway) or from a predator to its prey and their resources (top-down pathway). In directional terms, such pathways can be grouped into sink or source sub-webs, which include all the food chains originating from or converging to a single basal or top species respectively^24^. By propagating through feeding links, even small changes in species’ traits and single extinction or invasion events can significantly impact ecological communities^13,25^. Nevertheless, despite the importance of top-down and bottom-up controls on the dynamics of populations and ecological processes^19,20,24,26^, the mechanisms determining the distribution of species in sink and source sub-webs, as well as the relationship between species richness and the proportion of species sharing food chains in real food webs, have yet to be fully explored.

**Figure 1:**
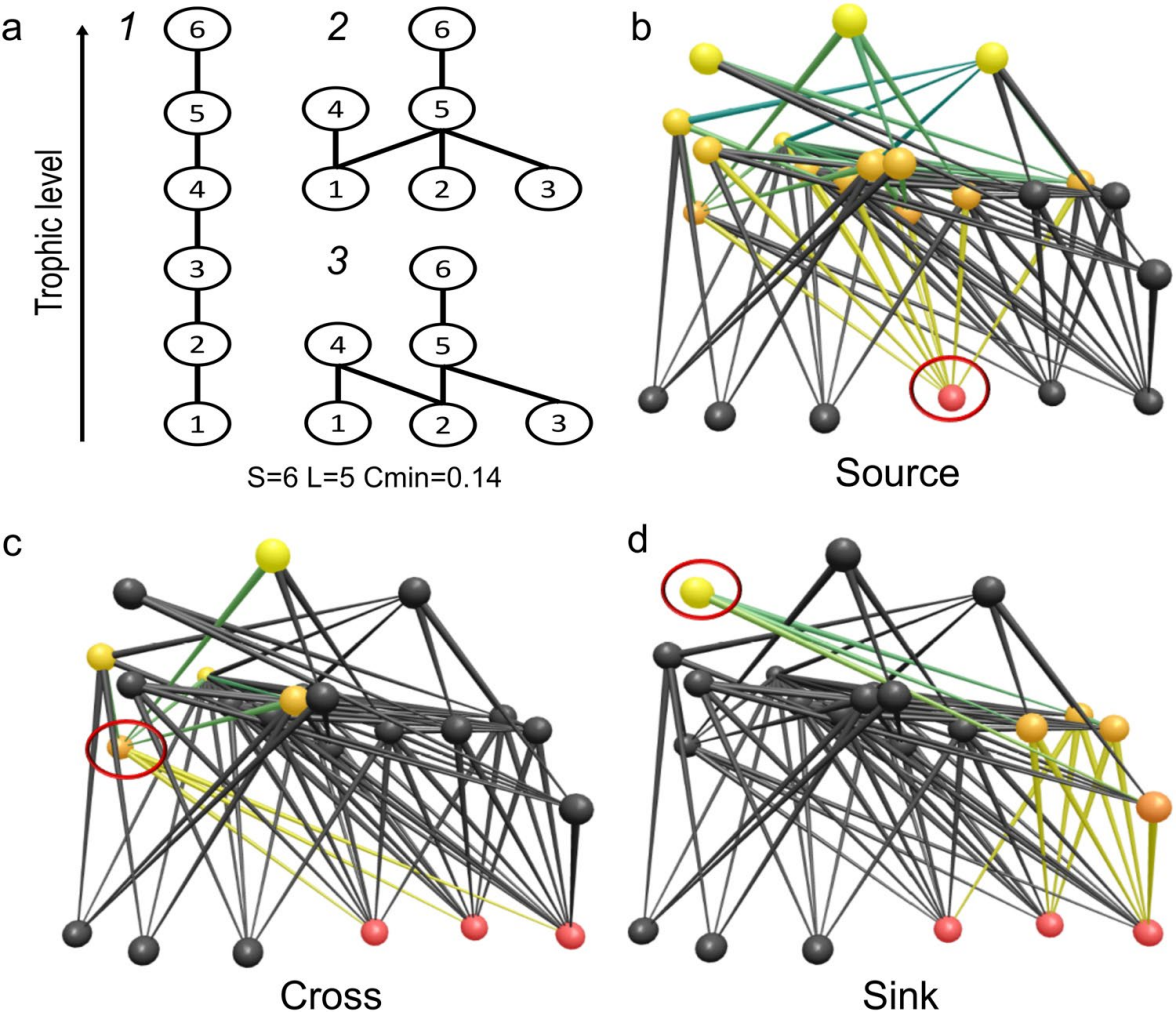
Panel (a) Comparison of three simple networks containing the same number of species (nodes) and trophic links (lines). In network 1, all species are part of one food chain, and a disturbance originating from any given species would directly propagate along the food chain to all the remaining ones. In network 2, a bottom-up disturbance spreading from species 1 would affect species 4-5-6 only. In network 3, the modification of a single link with respect to network 2 would mean that only species 4 would be directly affected by a bottom-up disturbance propagating from species 1. Panels (b–d) Food sub-webs exemplifying various propagation pathways along food chains for disturbance starting from a single species (circled): (**b**) bottom-up propagation from a basal resource (red), (**c**) cross propagation from a primary consumer (orange), and (**d**) top-down propagation from a predator (yellow).

We investigated the relationships between species richness, food web complexity and the distribution of species into source and sink sub-webs. We also considered a third type, the cross sub-web, which involves disturbance propagating in both directions from an intermediate species (Fig. 1). These represent fundamental aspects of biodiversity organisation, and underlie the potential of disturbance to propagate across trophic levels^13,24,25,27,28^. Accordingly, we assumed that the higher the proportion of species co-present in at least one food chain within a food web, the higher the potential of disturbance to propagate^13^. The intrinsic vulnerability of food webs to the propagation of disturbance along food chains was thus quantified as the proportion of species (P) included in each source (P_B_), cross (P_C_) and sink (P_T_) sub-web, originating from each basal, intermediate or top species respectively. Here, we focused on detritus-based food webs. The detritus compartment plays a fundamental role in ecosystem structure and functioning^29,30^, and climate change is expected to affect detritus inputs and organic matter decomposition rates in ecosystems^31,32^. Nevertheless, very little information that might help to understand the structure of these donor-controlled systems is available.

We compared food webs belonging to both aquatic and terrestrial habitats in the Mediterranean region (Table 1), and we quantified their vulnerability to disturbance propagating from species on various trophic levels. We then sought to verify whether the observed P_B_, P_C_ and P_T_ values were (i) dependent on species richness (S) and web connectance (Cmin) and (ii) predictable using a mechanistic food web model^6^ or similar to what would be expected by chance. This was achieved by comparing field data with random food web models, as well as webs generated using the niche model^6^, a simple yet predictive food web model based on observed S and Cmin values and a few foraging rules constraining the probability of feeding interaction between species. The comparison of observed and random food web structures can provide important insight into the organisation of ecological communities. Indeed, the association of food web stability with non-random structures enhances our understanding of the ecological factors constraining the observed patterns and hence the mechanisms underlying the emergence and persistence of complex food webs^7^.

**Table 1:** Food webs representative of various habitats in the Mediterranean region. Locality indicates the region, country and GPS coordinates for each habitat.

Habitat	Locality	S	Cmin	L/S
Lagoon	Sardinia (IT) 39°13′N 09°03′E	37	0.075	2.76
Stream, upstream	Lazio (IT) 41°51′N 13°00′E	45	0.073	3.30
Stream, downstream	Lazio (IT) 41°31′N 13°24′E	32	0.094	3.00
Lake	Lazio (IT) 42°05′N 12°12′E	28	0.130	3.65
Corn field	Catalunya (SP) 41°38′N 00°35′E	27	0.111	3.00
River, upstream	Lazio (IT) 41°58′N 12°30′E	21	0.114	2.39
River, downstream	Lazio (IT) 41°49′N 12°25′E	25	0.111	2.78
Beech forest	Lazio (IT) 42°19′N, 12°10′E	34	0.106	3.59

S: number of species in the food web; Cmin: food web connectance; L/S: species’ feeding linkage density.

## Results

### Species richness and food sub-webs

**Figure 2:**
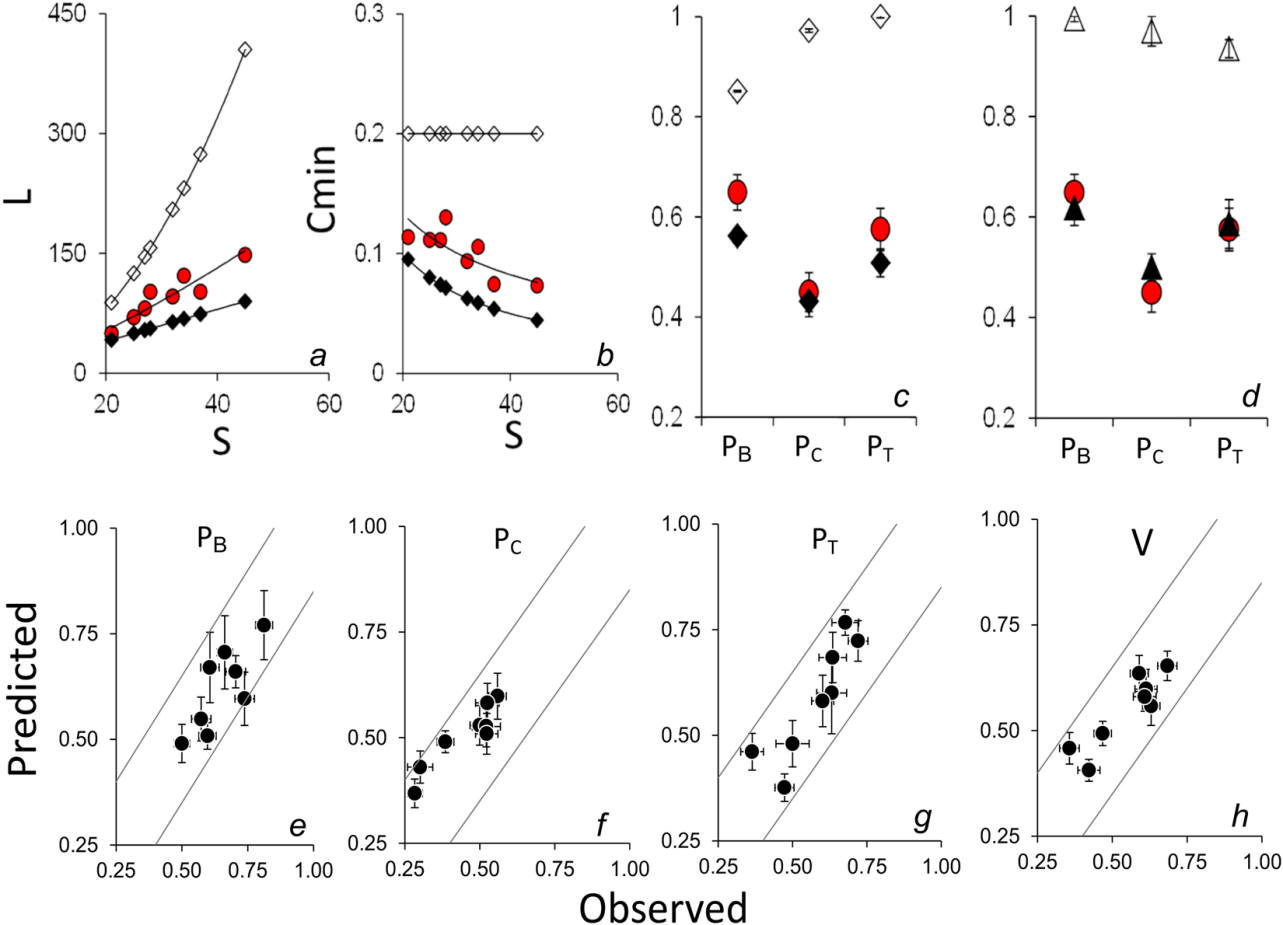
Panels (a,b) relationship between species richness (S), number of feeding links (L) and food web connectance (Cmin) observed in our dataset (red symbols) and estimated from the link-species scaling law (black symbols) and the constant connectance hypothesis (empty symbols). Panels (c,d) observed mean P_B_, P_C_ and P_T_ values (red symbols) in comparison with values estimated from the link-species scaling law (black diamonds) and the constant connectance hypothesis (empty diamonds), as well as values measured in model food webs generated using the niche model (black triangles) and a random model (empty triangles). P represents the proportion of species in each source (P_B_), cross (P_C_) or sink (P_T_) food sub-web. Lower panels (e–h) relationship between values observed in real food webs and those measured in niche model-generated webs. V represents the mean Pi value of all nodes in the food web. Grey lines represent y = x ± 0.15. Given that the maximum possible value of P is 1, the grey lines represent a ± 15% deviation from y = x.

**Figure 3:**
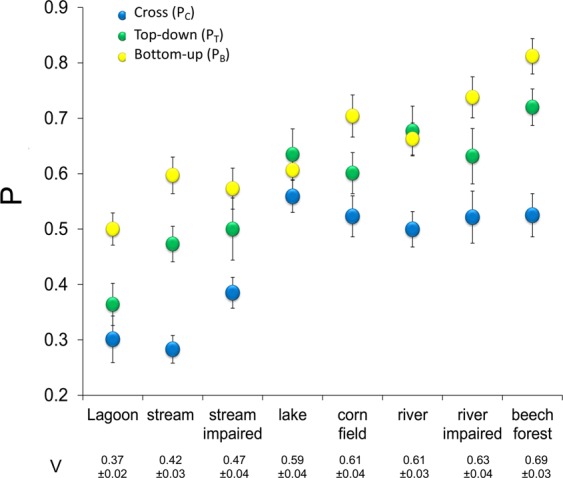
Comparison of the proportion of species (P) in the three types of disturbance propagation pathway. P_B_, P_C_ and P_T_ represent the mean proportion of species belonging to source, cross and sink sub-webs respectively. Food webs are ranked in order of V value, which represents the mean P value of all species within each food web.

**Figure 4:**
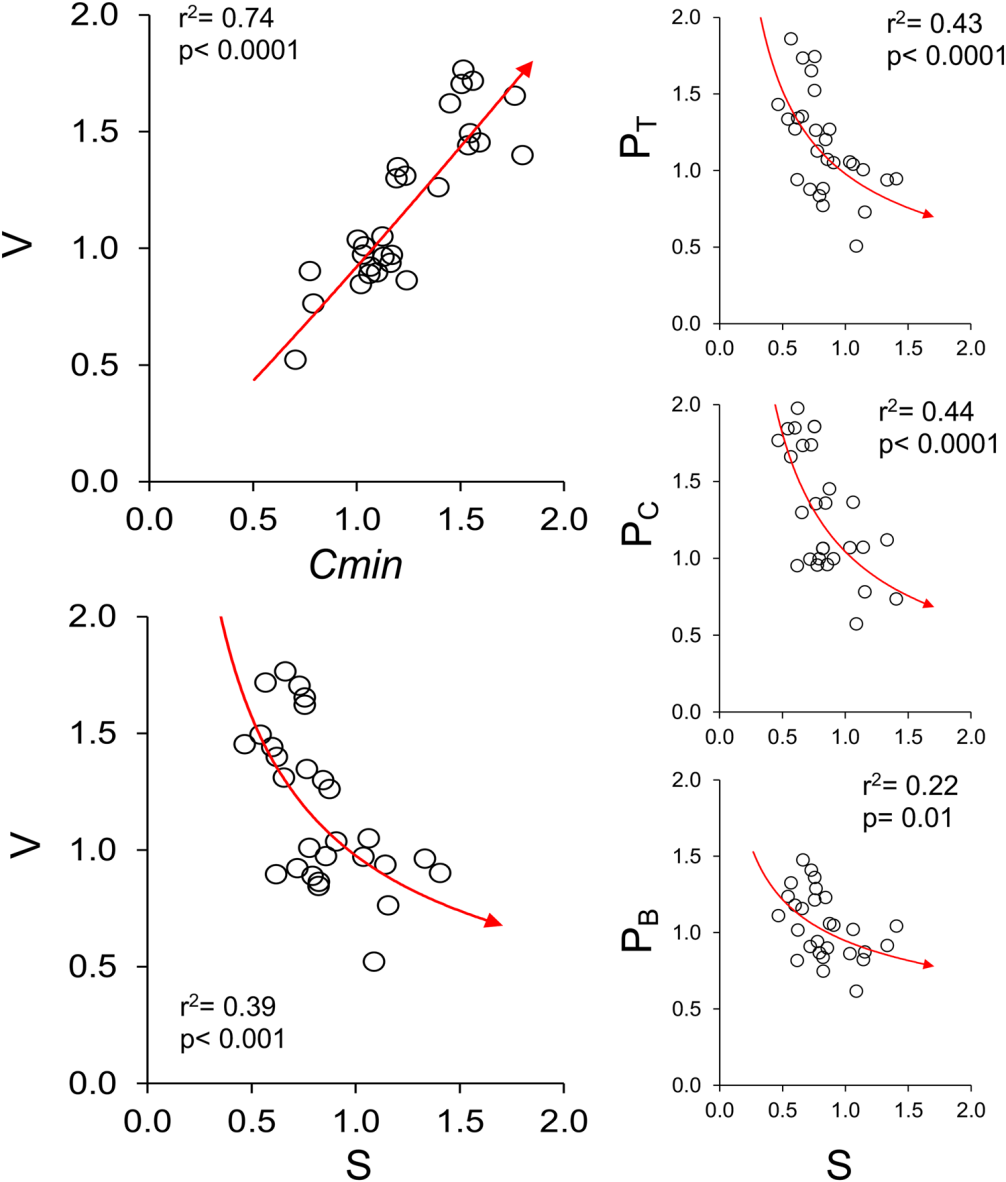
Correlations between the number of species (S), food web connectance (Cmin) and food web vulnerability to bottom-up (B), cross (C) and top-down (T) propagation of disturbance. Habitat pairwise ratios were calculated for each parameter, and the values were plotted (n = 28) and used for correlation models. Correlation models (red lines) and associated statistics are shown when robust to permutation (i.e. with a permutation-based p value < 0.05).

Food web connectance (Cmin) varied from 0.073 in Stream to 0.130 in Lake (Table 1), decreasing with species richness (S) (Fig. 2). On average, bottom-up propagation pathways (P_B_) included a higher proportion of species than cross (P_C_) and top-down (P_T_) propagation pathways (Fig. 3; one-way ANOVA for repeated measures: F = 36.7, p < 0.0001; associated Tukey’s pairwise comparisons: Q always > 4.4, p always < 0.01). P_B_, P_C_, P_T_ and V values differed between habitats (Fig. 3), all of them increasing with Cmin, while decreasing with S and intermediate species as a percentage of the total (%I) (Table 2 and Fig. 4). In contrast, P_B_, P_C_ and P_T_ were not related to the proportion of basal or top species (p always > 0.05). No differences in the correlation slopes between P_B_, P_C_ and P_T_ and either S or %I were observed (ANCOVA and the homogeneity of slopes test, F < 1 and p > 0.05 in both cases). In contrast, the correlation slope between P_B_ and Cmin was lower than what was observed for P_C_ and P_T_ (ANCOVA and homogeneity of slopes test, F = 4.5 and p = 0.01).

**Table 2:** Multiple regression models testing the effect of number of species (S), intermediate species as a percentage of the total (%I) and food web connectance (Cmin) on food web vulnerability to bottom-up (P_B_), cross (P_C_) and top-down (P_T_) propagation of disturbance across trophic levels, as well as on overall vulnerability to disturbance propagation (V).

Overall MANOVA						
	Wilks lambda	F	df1	df2	p	
	0.00	117.3	12	55.85	**4.77E-35**	
S	0.27	13.93	4	21	**1.07E-05**	
**Tests on independent variables**						
%I	0.02	255.7	4	21	**1.74E-17**	
Cmin	0.02	247.8	4	21	**2.40E-17**	
**Tests on dependent variables**						
	**R**^**2**^	**F**	**df1**	**df2**	**p**	
P_B_	0.98	440.5	3	24	**4.14E-21**	
P_C_	0.97	229.5	3	24	**8.47E-18**	
P_T_	0.94	115.6	3	24	**2.10E-14**	
V	0.99	891.9	3	24	**9.78E-25**	
**Regression coefficients and statistics**						
		**Coeff**.	**Std.err**.	**t**	**p**	**R**^**2**^
P_B_	S	−0.20	0.03	−6.37	**1.40E-06**	0.22
	%I	−0.60	0.02	−28.40	**5.55E-20**	0.91
	Cmin	0.35	0.04	9.71	**8.67E-10**	0.38
P_C_	S	−0.08	0.06	−1.19	**2.45E-03**	0.44
	%I	−0.24	0.04	−5.43	**1.41E-05**	0.38
	Cmin	1.23	0.07	16.54	**1.27E-14**	0.92
P_T_	S	−0.22	0.08	−2.68	**1.30E-03**	0.43
	%I	−0.52	0.06	−9.46	**1.45E-09**	0.65
	Cmin	0.90	0.09	9.45	**1.46E-09**	0.69
V	S	−0.18	0.03	−5.86	**4.86E-06**	0.39
	%I	−0.54	0.02	−26.23	**3.54E-19**	0.69
	Cmin	0.88	0.04	25.25	**8.51E-19**	0.74

Bold values indicate a significant effect, with a p value < 0.05.

**Figure 5:**
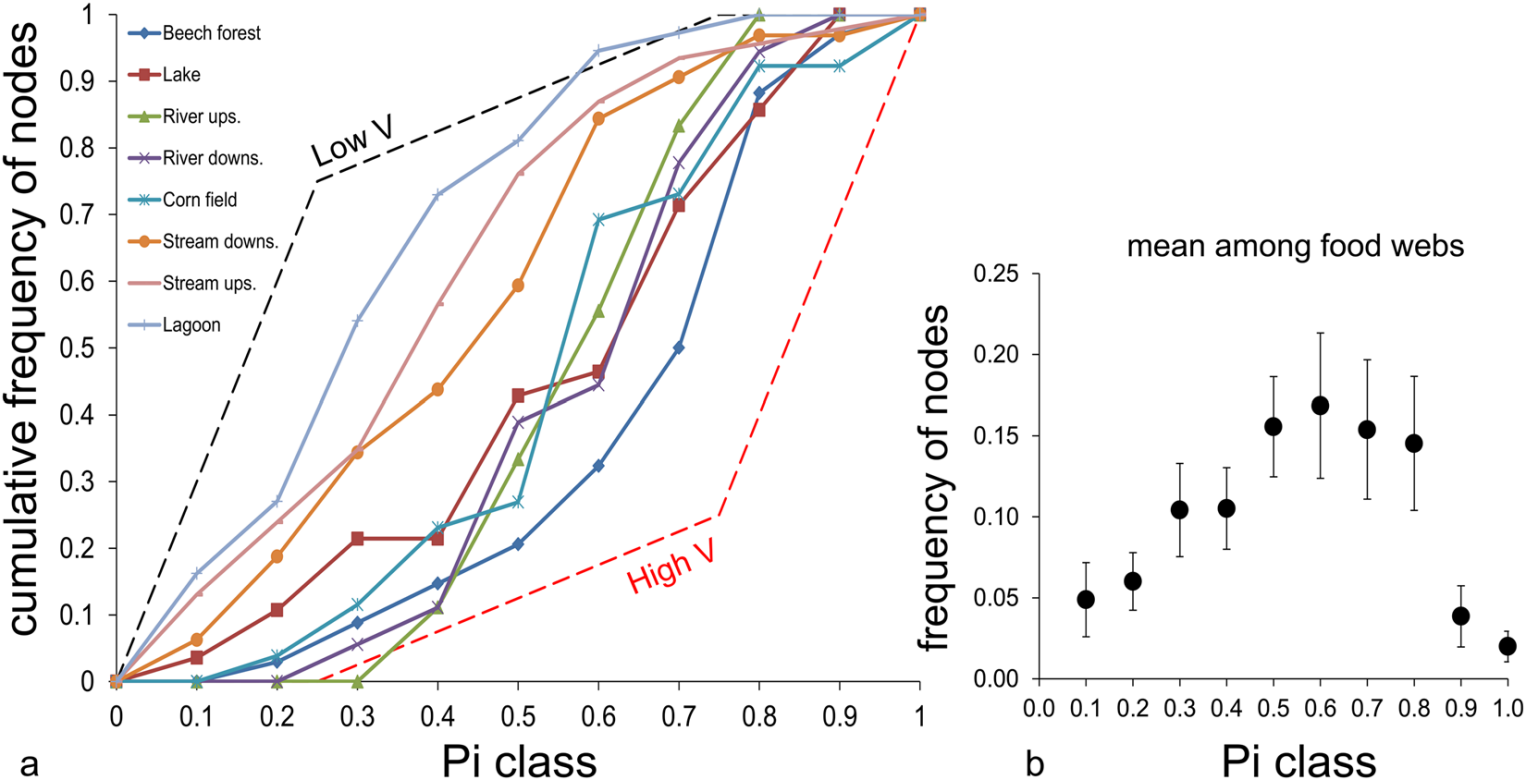
(**a**) Cumulative frequency of nodes (i.e. species) by P value (for P classes of 0.1). Black and red dashed lines represent reference thresholds identifying food webs with low vulnerability (species with P < 0.25 being relatively more numerous, and no species with P > 0.75) or high vulnerability (species with P > 0.75 being relatively more numerous, and no species with P < 0.25) to disturbance propagation (V). (**b**) mean ( ± standard error among food webs) proportion of species within each class.

On average, species having a high or low Pi value (i.e. species sharing at least one food chain with a markedly high or low proportion of remaining species in the food web) were infrequent (Fig. 5). The variability (i.e. the coefficient of variation, c.v.) of the Pi values of species within each food web increased with S and %I (Table S2), and was inversely related to P_B_, P_C_, P_T_ and V (Fig. S1, Overall statistics and MANOVA: r^2^ = 0.56, Wilks’ lambda = 0.32, F = 12.2, p = 6.2E^−05^. Linear regressions: r^2^ > 0.48 and p < 6.7E^−05^ in all cases; ANCOVA and homogeneity of slopes test, F = 0.66, p > 0.05).

### Observed vs. model food webs

The correlation slope between observed S and Cmin was consistent with expectations from the link-species scaling law^33^ (LSSL) (Fig. 2a,b) (One-way ANCOVA and homogeneity of slopes test, F = 0.25, p = 0.63), which predicts that L/S will not be dependent on S, L will increase as L = 2S^1^ and Cmin will scale with S. As a consequence, the observed P_B_, P_C_ and P_T_ values were similar to those expected on the basis of the LSSL (Fig. 2c), while they were significantly lower than what would be expected on the basis of the constant connectance hypothesis^34^ (CCH) (Two-way ANOVA and Tukey’s pairwise comparisons, p < 0.05), which predicts that L/S will increase with S, L will increase as L = 0.2S^2^ and Cmin will not vary with S (Fig. 2c). Lastly, starting from the observed S and Cmin values, we generated model food webs based on the niche model^6^, which were then compared with random food web models. The niche model-generated webs closely predicted the mean and habitat-specific P_B_, P_C_, P_T_ and V values observed in the real food webs (Fig. 2d-h). In contrast, the random model-generated webs were characterised by markedly higher values (Fig. 2d, one-way ANOVA for repeated measures and Tukey’s pairwise comparisons, p always < 0.05).

## Discussion

The distribution of species across source, cross and sink sub-webs differed between habitats in a predictable way in accordance with food web size (S) and connectance (Cmin). The higher the number of species, the lower the proportion of them co-present in at least one food chain. This indicates that the vulnerability of ecosystems to disturbance propagation across trophic levels falls as their species richness rises, which implies that more biodiverse communities are potentially more able to buffer disturbance. Our observations are also consistent with recent results from studies based on model food webs of similar species richness^13^, where higher connectance intensified the effect (i.e. caused a higher rate of secondary species loss) of single perturbation events (i.e. successful species invasions). As well as species richness, a high number of intermediate species relative to the total reduced the potential of disturbance to propagate along food chains. In addition, our data satisfied expectations from the link-species scaling law, which predicts constant linkage density among species. According to foraging optimisation theories, (i) a low and roughly constant linkage density between species arises from the selection by consumers of the lowest number of food items that maximises their net energy intake^9,35^, and (ii) prey species richness promotes predator trophic specialisation, as shown in the food webs analysed here^32,36^ and elsewhere^9,37^. This suggests that energetic constrains on foraging strategies, which operate at the individual level^38^, may give rise to food webs which are intrinsically less vulnerable to the propagation of disturbance than what would be expected if consumers tended to generalise as the number of their potential food sources increased^39^. Notably, the random food web models failed to reproduce real patterns, while niche model-generated webs closely predicted the observed values. Our results thus imply that the distribution of species in source, cross and sink sub-webs significantly deviates from random, and that the patterns observed in real food webs benefit ecological communities by reducing vulnerability to disturbance propagation along food chains compared to what would be expected if distribution was determined by chance.

Regardless of habitat type, source sub-webs included a higher proportion of total species than cross and sink sub-webs. This implies that disturbance propagating from a basal resource would have a significantly higher potential to spread throughout the food web than disturbance propagating from a top or an intermediate species. We speculate that the observed patterns may promote stability in detritus-based systems, where the availability of basal resources (i.e. organic detritus and colonising microfungi) is expected to be much more stable over time than invertebrate populations, often characterised by marked spatial-temporal variations and sensitivity to stress^40,41^ (see Fig. S2 for supporting results). On the other hand, global change is expected to affect the dynamics of the detritus compartment in ecosystems^31,32,42,43^. Thus, our data suggest that there is a strong likelihood of future environmental changes in Mediterranean ecosystems giving rise to bottom-up effects mediated by the structure of detritus-based food webs.

The coefficient of variation of Pi values increased with the number of species within each food web, and it was inversely related to food web vulnerability to disturbance. Together with the P_B_, P_C_, P_T_ and V values, this makes the variation of species’ Pi values within a food web a useful a-priori indication of its vulnerability. We acknowledge that our observations are based on static food web structures, and that the reorganisation of trophic links and/or changes in linkage strength between species in accordance with their trophic niche plasticity could modify the effects of disturbance^25^. Nevertheless, our analysis of sub-webs enabled us to evaluate the intrinsic vulnerability of real food webs to a range of disturbance propagation pathways (i.e. bottom-up, cross, and top-down). This approach is expected to yield information useful to management strategies based on the risk of ecosystem-specific disturbance propagation, regardless of food web compilation methods. Analysis of the scaling relationships between the size of networks and that of the sub-webs of which they are composed could also be extended to non-natural systems (e.g. financial^44^, transport^45^ or disease vector dispersal^46^ networks). This would make it possible to verify whether (i) systemic risk increases with network size or (ii) the rules governing such systems mean that an increase in size is accompanied by increased potential to buffer disturbance, as observed in the food webs analysed in this study.

In conclusion, we show that increasing species richness and foraging constraints on food web complexity limit the proportion of species that share food chains in ecosystems. This pattern has not been noticed before. It reduces the intrinsic vulnerability of biodiverse ecological communities to disturbance propagation, and promotes the emergence of species-rich food webs where species organise into effective disturbance-buffering structures in accordance with predictable rules. It is also consistent with the predictions of theoretical research, which highlight the positive effect of adaptive trophic behaviour by consumers on the stability of model food web networks^9^. Conversely, the observed results imply that species-poor ecosystems could be highly vulnerable to the propagation of disturbance along food chains, including bottom-up disturbance associated with climate change^31,42^, with increased risks for the stability of food webs and the ecosystem services they support^47–49^. While this study focused on detritus-based food webs, the observed results may be extended to more complete food webs including primary producer-based food chains. Indeed, detrital and herbivore energy pathways are closely interconnected^8^, and our observations are consistent with webs generated using the niche model, which was developed and tested against whole food web structures in both aquatic and terrestrial ecosystems^6^. Notably, our food web analysis provides mechanism-based evidence indicating that efforts devoted to biodiversity conservation also increase the potential of natural communities to buffer disturbance in ecosystems by maintaining biodiverse, relatively less complex, and ‘safer’ food web structures for their constituent species.

## Materials and Methods

We compared habitat-scale detritus-based food webs (Table 1), reconstructed using population abundance data, carbon and nitrogen stable isotope values and Bayesian isotopic mixing models (for details on stable isotope analyses and food web reconstruction methods, see Rossi *et al*.^32^ and Calizza *et al*.^12,36^). Specifically, the habitat-scale food webs were based on taxa and trophic links obtained by sampling multiple patches within each habitat. This made it possible to taking into account intra-habitat variability of occurrence data and the trophic preferences of the various taxa^12^. The food webs were representative of six different habitats: a river flowing from a semi-natural to an anthropic landscape (the River Sacco, referred to as Stream), a lowland high-order river embedded in an urban landscape crossing the city of Rome (the River Tiber, referred to as River), a meso-oligotrophic lake (Lake Bracciano, referred to as Lake); a transitional water system (the Santa Gilla lagoon, referred to as Lagoon), arable land (referred to as Corn Field) and a woodland (referred to as Beech Forest) (Table 1). The lower stretches of the two river courses were more heavily anthropised and barriers were present in both cases (e.g. the city of Rome along the River Tiber). Therefore, River and Stream were each divided into upstream and downstream habitats. In all habitats, basal resources were represented by habitat-specific leaf litter colonised by various species of microfungi^26,50^. Detailed descriptions of sampling activities and habitats can be found in Rossi *et al*.^32^ (Lake, Lagoon, Beech Forest, Corn Field), in Calizza *et al*.^12,36^ (River), and in Bentivoglio *et al*.^51^ (Stream).

Web topology was analysed by determining (1) species richness, S; (2) the number of feeding links, L; (3) linkage density, L/S; (4) connectance (Cmin) as L/S^2^; and (5) the proportions of total species in the food web accounted for by basal (%B), intermediate (%I) and top (%T) species. %I was considered to be a measure of prey availability for predators^32,36^. The intrinsic vulnerability of food webs to bottom-up, cross and top-down propagation of disturbance (i.e. starting from basal, intermediate and top species) was quantified as the proportion of the total number of species (P) occurring in each source (P_B_), cross (P_C_) and sink (P_T_) sub-web respectively^24^ (Fig. 1), as follows:

$${\rm{P}}=(\mathop{\sum }\limits_{i=1}^{nx}{p}_{i})/nx$$

where nx is the number of basal, intermediate and top species used to calculate P_B_, P_C_ and P_T_ respectively, *p*_i_ = Si/(S-Ni), Si = the number of species included in the sub-web originating from the i^th^ species, S = the total number of species in the food web, and Ni = number of species on the same trophic level as the i^th^ species. The overall vulnerability of the food web to disturbance propagation along food chains (V) was measured as the mean P value of all species in the food web.

For each parameter, pairwise ratios between habitats were calculated and a matrix containing pairwise comparisons was created. Pairwise values (n = 28) were plotted and used for correlation models^52,53^. A permutation test (9999 permutations) was also run on correlation coefficients, and the observed correlations were considered significant only if robust to permutation (i.e. with a permutation-based p value < 0.05). Differences in correlation slopes were tested by analysis of covariance (ANCOVA) and the associated homogeneity of slopes test.

Based on the observed S and Cmin values, for each habitat, 20 model food webs were generated. Specifically, 10 model food webs were generated based on the niche model^6^, a simple yet predictive one-dimensional model able to reproduce the topological properties of real food webs starting from a small number of rules governing the probability of feeding interaction between species. The other 10 model food webs were generated by assigning links between species at random. The P_B_, P_C_, P_T_ and V values were calculated and compared with the values measured in the real food webs. In addition, the L and Cmin values expected in accordance with the link-species scaling law^33^ (L_LSSL_, Cmin_LSSL_) and the constant connectance hypothesis^34^ (L_CCH_, Cmin_CCH_) were calculated with reference to the observed S value for each habitat. Using the correlation coefficients obtained from real webs, the P_B_, P_C_ and P_T_ values were then extrapolated from the correlation with either Cmin_LSSL_ or Cmin_CCH_ and compared with the observed data. Food web analysis and modelling were performed with FoodWeb3D software.

## Supplementary information

Supplementary material
