# Supplementary material for: Species richness and vulnerability to disturbance propagation in real food webs
Source: Sci Rep. 2019 Dec 18;9:19331. doi: 10.1038/s41598-019-55960-8 (PMC6920442; doi:10.1038/s41598-019-55960-8)
Supplement: Supplementary file 1 — Supplementary material [file 41598_2019_55960_MOESM1_ESM.pdf]

**SUPPLEMENTARY MATERIAL**

# Species richness and vulnerability to disturbance propagation in real food webs

Edoardo Calizza[1,2,*], Loreto Rossi[1,2], Giulio Careddu[1], Simona Sporta Caputi[1], Maria Letizia Costantini[1,2]

[1]Department of Environmental Biology, Sapienza University of Rome, Via dei Sardi 70, 00185 Rome, Italy.

[2]National Inter-University Consortium for Marine Sciences (CoNISMa), Piazzale Flaminio 9, 00196, Rome, Italy.

*Corresponding Author: edoardo.calizza@uniroma1.it

## Supplementary material contents

**Table S1:** Variability of Pi values within each habitat.

**Table S2:** Effect of the total number of species (S), intermediate species as a percentage of the total (%I) and food web connectance (Cmin) on the variability of Pi values within each food web.

**Fig. S1:** Correlation between variability of Pi values within each food web and vulnerability to disturbance propagation.

**Fig. S2:** Intra-habitat variation of organic content in sediment and abundance of various species.

## SUPPLEMENTARY TABLES

**Table S1. a**: Coefficient of Variation (C.V.) of the Pi value of nodes within each food web. **b**: pairwise comparisons of C.V. values, Fligner-Killeen test[53]. Numbers in the matrix represent the p value for each pairwise comparison. n.s. means a p value > 0.10.

| **a** | C.V. | 51.9 | 45.0 | 32.0 | 48.8 | 27.3 | 20.4 | 24.7 | 38.4 |
|---|---|---|---|---|---|---|---|---|---|
| **b** | | Stream ups. | Stream downs. | Corn field | Lagoon | Beech forest | River ups. | River downs. | Lake |
| Stream ups. | | | n.s. | 0.05 | n.s. | 0.01 | 0.01 | 0.03 | n.s. |
| Stream downs. | | | | 0.10 | n.s. | 0.03 | 0.01 | 0.05 | n.s. |
| Corn field | | | | | 0.02 | n.s. | 0.07 | n.s. | n.s. |
| Lagoon | | | | | | 0.02 | 0.01 | 0.02 | n.s. |
| Beech forest | | | | | | | n.s. | n.s. | n.s. |
| River ups. | | | | | | | | n.s. | 0.04 |
| River downs. | | | | | | | | | n.s. |
| Lake | | | | | | | | | |

**Table S2**. Multiple regression models and associated coefficients and statistics testing the effect of the number of species (S), intermediate species as a percentage of the total (%I) and food web connectance (Cmin) on the variability (i.e. C.V.) of Pi values within each food web. Bold values indicate a significant effect (p < 0.05).

| | | | | | | |
|---|---|---|---|---|---|---|
| | | | Overall MANOVA | | | |
| | Wilks lambda | F | df1 | df2 | p(regression) | |
| | 0.01 | 72.3 | 3 | 24 | **3.67E-12** | |
| | | | Tests on independent variables | | | |
| | Wilks lambda | F | df1 | df2 | p | |
| S | 0.368 | 41.17 | 1 | 24 | **1.23E-06** | |
| %I | 0.224 | 83.16 | 1 | 24 | **2.88E-09** | |
| Cmin | 0.912 | 2.317 | 1 | 24 | 0.14 | |
| | | | Tests on dependent variables | | | |
| | R^2 | F | df1 | df2 | p | |
| C.V. | 0.90 | 72.3 | 3 | 24 | **3.67E-12** | |
| | | | Regression coefficients and statistics | | | |
| | | Coeff. | Std.err. | t | p | R^2 |
| C.V. | S | 0.98 | 0.15 | 6.42 | **1.23E-06** | 0.55 |
| | %I | 0.75 | 0.08 | 9.12 | **2.88E-09** | 0.69 |
| | Cmin | 0.19 | 0.13 | 1.52 | 0.14 | 0.29 |

**SUPPLEMENTARY FIGURES**

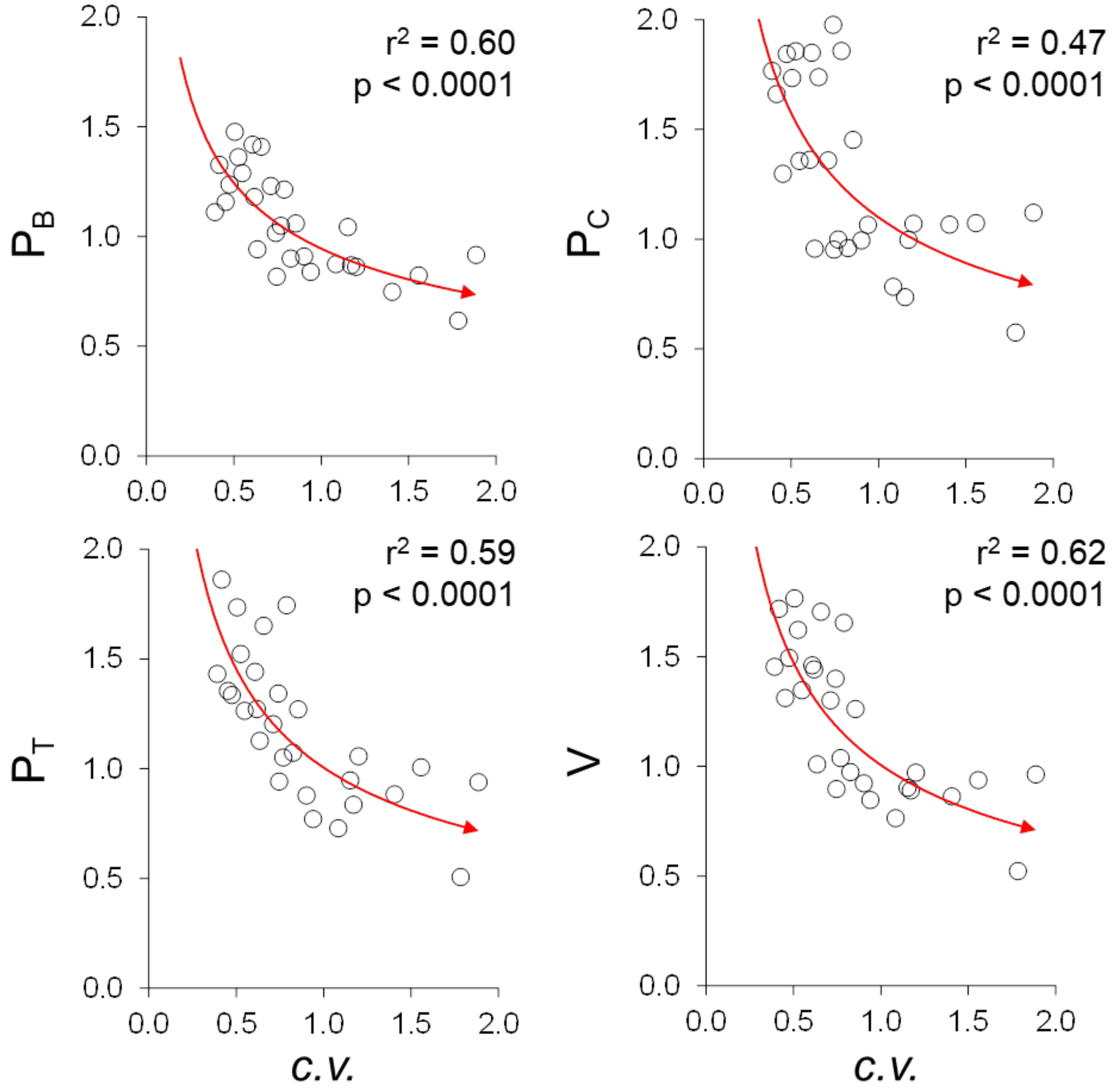


**Fig. S1.** Correlation between the variability (C.V.) of species' Pi values within each food web and vulnerability to bottom-up ($P_B$), cross ($P_C$) and top-down ($P_T$) propagation of disturbance across trophic levels, as well as the overall vulnerability of the food web to disturbance propagation (V). Pairwise ratios between habitats were calculated for each parameter, and the values were plotted (n = 28) and used for regression models. Regression models (red lines) and associated statistics are shown when robust to permutation (i.e. with a permutation-based p value < 0.05).

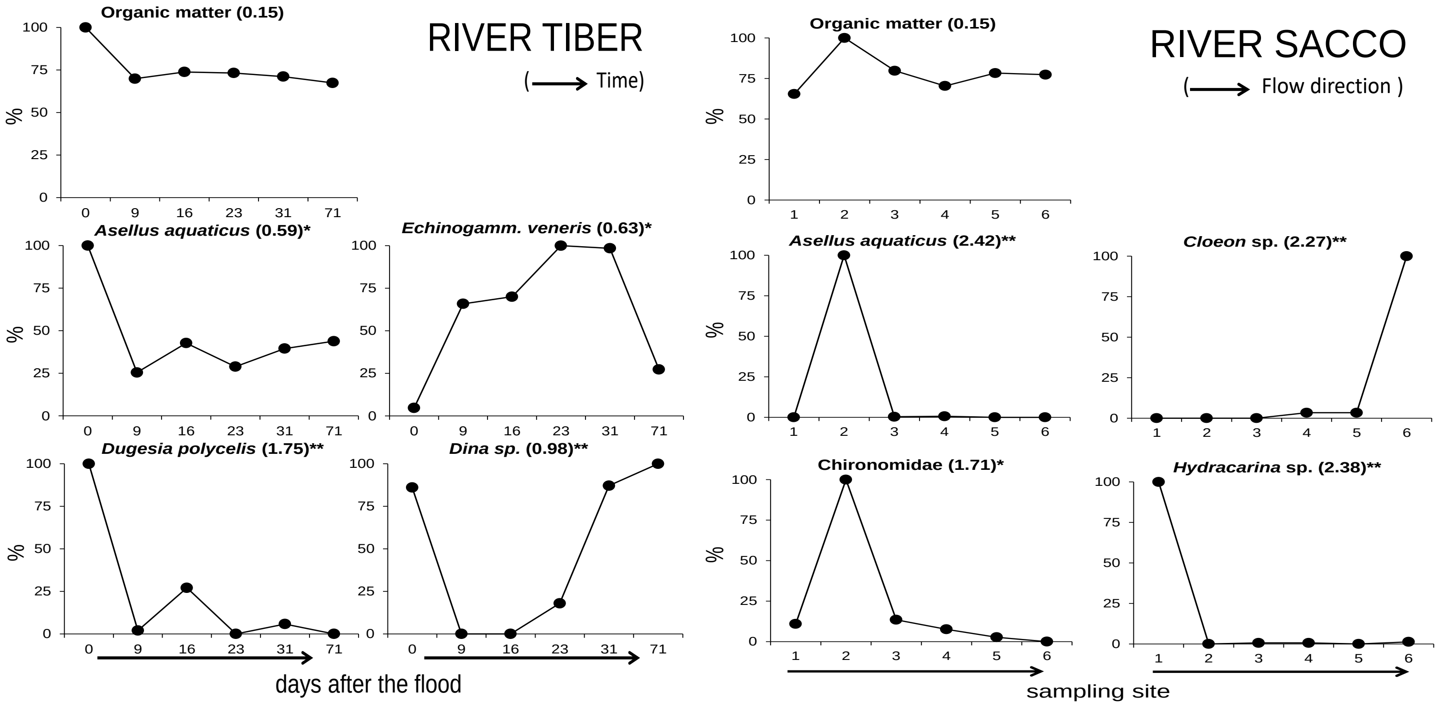


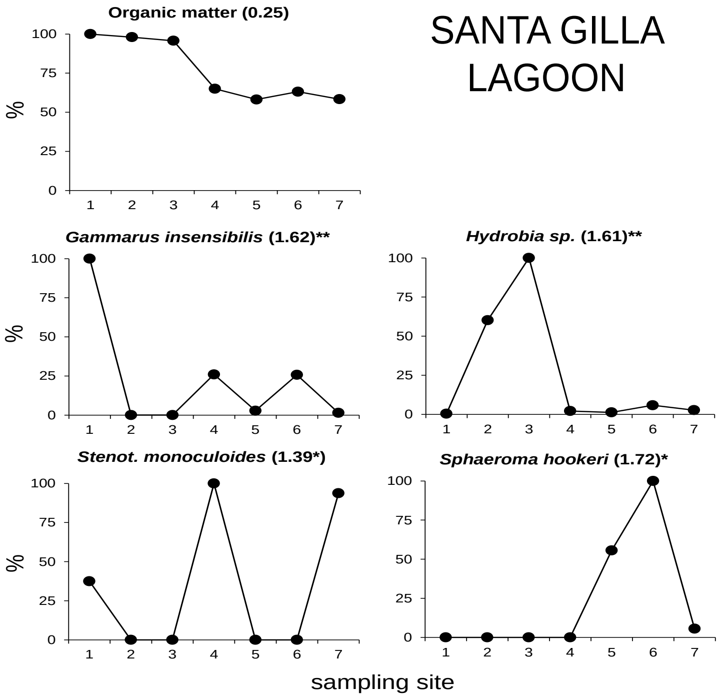


**Fig. S2.** Variation (with respect to the maximum observed value within each habitat, indicated as 100%) of organic content in sediment (Organic matter) and abundance of selected invertebrate species in three habitats. The selected species represent relatively abundant species found in at least 50% of sampling sites within each habitat. Variations are expressed over time, i.e. across six consecutive sampling times in the River Tiber, and space, i.e. across 6 and 7 spatially distinct sampling sites in the River Sacco and Santa Gilla Lagoon respectively. The River Sacco sites run from upstream (site 1) to downstream (site 6). Numbers in parentheses are the coefficients of variation (C.V.) of organic matter content and species density across sampling times/sites. Asterisks indicate a significantly higher C.V. in invertebrates than organic matter in sediment (Fligner-Killeen test, $p < 0.05$).
